# Cases in Human Parasitology

**DOI:** 10.3201/eid1105.050203

**Published:** 2005-05

**Authors:** Mark L. Eberhard

**Affiliations:** * Centers for Disease Control and Prevention, Atlanta, Georgia, USA

**Keywords:** Parasitology, Cases in, diagnostics

This compact, glossy paperbound text contains 62 cases in 5 sections: I) Intestinal Protozoa, II) Blood and Tissue Protozoa, III) Cestodes, Trematodes and Intestinal Nematodes, IV) Blood and Tissue Nematodes, and V) Challenging Cases. The intent was to emphasize the relationship between diagnosis and patient care. This goal is laudable; unfortunately, serious shortcomings limit the book's usefulness for students, professors, or laboratorians.

A major challenge is the format: the presented case is "textbook," and the "answer" is given in the case presentation, leaving little need for the answer section. How many junior parasitologists or biologists with casual interest in parasites don't know that bile-stained, barrel-shaped nematode eggs with prominent polar plugs represent *Trichuris* eggs? If the clinical history and illustrations are presented and the detailed description of the organism is left to the answer, the reader can look at the illustrations, decipher the morphologic features, consider the possibilities, and then differentiate by using existing features.

Some case presentations had no illustrations, which is a prerequisite. Most illustrations were adequate, but some were unacceptable. Figure 4.1 presumes to illustrate an *Entamoeba histolytica* cyst, but the diagnosis could not be made from the image. Figure 16.1 is listed as typical of *Babesia* infection, yet after close study, if *Babesia* organisms are present, they are not typical. The illustrations of microsporidia at low-power magnification were perplexing. Use of identical images to illustrate East African and West African trypanosomes is unacceptable. The illustration for case 52 (onchocerciasis) shows a Giemsa-stained microfilaria with a sheath. The morphologic features and the sheath stained with Giemsa indicate a *Brugia* microfilaria, not an *Onchocerca* microfilaria.

In case 52 (*Onchocerca*), surgical removal of regional lymph nodes is advised, in addition to removal of nodules containing adult worms. This is not standard medical advice. In case 48 (dracunculiasis), it is stated that cisterns in Iran and step wells in India are common sources of infection and that prevalence of this infection has been reduced in most areas, except India, Pakistan, and a few countries in Africa. Guinea worm has been absent from Iran since 1972, from Pakistan since 1993, and from India since 1996. In the same case study, it is stated that metronidazole is often used to complement or replace traditional removal of worms, and that niridazole, thiabendazole, and mebendazole are also useful. None of these drugs has any benefit in Guinea worm infection treatment. In case 3 (cyclosporiasis), it is stated that infections from ingestion of contaminated fruits, such as imported strawberries, have been reported. Not true; strawberries have never been implicated.

Given the multiple errors and lack of attention to detail (Colombia is misspelled; the width of Anisakis L3 is given as 1 cm), this book has little to offer, despite its reasonable price ($60). This is unfortunate because a well-done series of teaching cases could fill a much needed void.

